# Exploring the prognostic value of BRMS1 + microglia based on single-cell anoikis regulator patterns in the immunologic microenvironment of GBM

**DOI:** 10.1007/s11060-024-04781-5

**Published:** 2024-08-15

**Authors:** Songyun Zhao, Kaixiang Ni, Jiaheng Xie, Chao Cheng, Ning Zhao, Jinhui Liu, Wei Ji, Qi Wang, Pengpeng Zhang, Yuankun Liu

**Affiliations:** 1 Department of Neurosurgery, The Affiliated Wuxi People’s Hospital of Nanjing Medical University, Wuxi, China; 2 Wuxi Medical Center of Nanjing Medical University, Wuxi, China; 3 Department of Plastic Surgery, Xiangya Hospital, Central South University, Changsha, China; 4 Department of Gynecology, The First Affiliated Hospital of Nanjing Medical University, Nanjing, China; 5 Department of Gastroenterology, Affiliated Hospital of Jiangsu University, Zhenjiang, China; 6 Department of Lung Cancer Surgery, Tianjin Lung Cancer Center, National Clinical Research Center for Cancer, Key Laboratory of Cancer Prevention and Therapy, Tianjin’s Clinical Research Center for Cancer, Tianjin Medical University Cancer Institute and Hospital, Tianjin, China

**Keywords:** Anoikis, scRNA-seq, stRNA-seq, GBM, NMF, M2 macrophage polarization, Tumor microenvironment

## Abstract

**Background:**

Anoikis is a specialized form of programmed cell death induced by the loss of cell adhesion to the extracellular matrix (ECM). Acquisition of anoikis resistance is a significant marker for cancer cell invasion, metastasis, therapy resistance, and recurrence. Although current research has identified multiple factors that regulate anoikis resistance, the pathological mechanisms of anoikis-mediated tumor microenvironment (TME) in glioblastoma (GBM) remain largely unexplored.

**Methods:**

Utilizing single-cell RNA sequencing (scRNA-seq) data and employing non-negative matrix factorization (NMF), we identified and characterized TME cell clusters with distinct anoikis-associated gene signatures. Prognostic and therapeutic response analyses were conducted using TCGA and CGGA datasets to assess the clinical significance of different TME cell clusters. The spatial relationship between BRMS1 + microglia and tumor cells was inferred from spatial transcriptome RNA sequencing (stRNA-seq) data. To simulate the tumor immune microenvironment, co-culture experiments were performed with microglia (HMC3) and GBM cells (U118/U251), and microglia were transfected with a BRMS1 overexpression lentivirus. Western blot or ELISA were used to detect BRMS1, M2 macrophage-specific markers, PI3K/AKT signaling proteins, and apoptosis-related proteins. The proliferation and apoptosis capabilities of tumor cells were evaluated using CCK-8, colony formation, and apoptosis assays, while the invasive and migratory abilities of tumor cells were assessed using Transwell assays.

**Results:**

NMF-based analysis successfully identified CD8 + T cell and microglia cell clusters with distinct gene signature characteristics. Trajectory analysis, cell communication, and gene regulatory network analyses collectively indicated that anoikis-mediated TME cell clusters can influence tumor cell development through various mechanisms. Notably, BRMS1 + AP-Mic exhibited an M2 macrophage phenotype and had significant cell communication with malignant cells. Moreover, high expression of BRMS1 + AP-Mic in TCGA and CGGA datasets was associated with poorer survival outcomes, indicating its detrimental impact on immunotherapy. Upregulation of BRMS1 in microglia may lead to M2 macrophage polarization, activate the PI3K/AKT signaling pathway through SPP1/CD44-mediated cell interactions, inhibit tumor cell apoptosis, and promote tumor proliferation and invasion.

**Conclusion:**

This pioneering study used NMF-based analysis to reveal the important predictive value of anoikis-regulated TME in GBM for prognosis and immunotherapeutic response. BRMS1 + microglial cells provide a new perspective for a deeper understanding of the immunosuppressive microenvironment of GBM and could serve as a potential therapeutic target in the future.

**Supplementary Information:**

The online version contains supplementary material available at 10.1007/s11060-024-04781-5.

## Introduction

The World Health Organization (WHO) classifies gliomas into grades I-IV based on histological and molecular characteristics. Grade IV glioma, also known as glioblastoma (GBM), is one of the most common and aggressive primary malignant brain tumors in adults, accounting for approximately 57% of all gliomas [1]. The current standard treatment paradigm for GBM, known as the Stupp regimen, requires maximal surgical resection of the tumor, followed by combined radiotherapy and chemotherapy [2]. Despite revolutionary advancements in treatment methods, the 5-year survival rate for GBM patients remains at only 5.8%, with nearly all patients experiencing recurrence after standard therapy [3]. In recent years, with a deeper understanding of the underlying molecular biology of GBM and its interaction with the immune system, there is a need to further refine treatment strategies and explore new approaches in the fields of immunotherapy and precision oncology [4].

Immunotherapy strategies have revolutionized the treatment of various cancers in recent years, yet the unique microenvironment of the central nervous system has long been considered immunologically privileged [3]. Immune checkpoint blockade (ICB) therapy has proven feasible and effective in treating many cancers; however, to date, there have been no successful phase III clinical trials or market approvals for ICBs in GBM treatment [5, 6]. Theoretically, immune cells can cross the blood–brain barrier and target infiltrating tumor cells, making GBM an ideal candidate for immunotherapy. However, GBM is a tumor that rarely elicits T-cell inflammatory responses and is associated with a high percentage of terminally differentiated CD8 + tumor-infiltrating lymphocytes (TILs), which are less responsive to ICBs [7]. Additionally, tumor-associated microglia and macrophages (TAMs) promote immunosuppressive effects by secreting various growth factors, angiogenic factors, and immunosuppressive cytokines, enhancing T-cell apoptosis and rendering it an "immunologically cold" tumor [8].

Anoikis is a specialized form of programmed cell death that occurs when cells lose interaction with the surrounding extracellular matrix (ECM) [9]. The triggering mechanism of anoikis involves the separation of cells from the ECM, leading to the loss of integrin-mediated survival signals, activation of Bcl-2 modifiers, and ultimately inducing anoikis [10]. Although anoikis plays a crucial role in maintaining tissue homeostasis, in diseases such as cancer, cells can occasionally evade death signaling pathways and develop anoikis resistance. The emergence of anoikis resistance allows detached cells to bypass death signaling pathways, enabling them to survive under adverse conditions [11]. Current research primarily focuses on exploring the potential regulatory mechanisms of anoikis through various endogenous and exogenous pathways, such as integrins, epidermal growth factor receptor (EGFR), TGF-β signaling, NF-κB signaling, and hypoxia [12]. Recent findings suggest that regulatory signals activated by β1 integrin play a key role in the acquisition and development of anoikis resistance in GBM cells [13]. Therefore, investigating the expression of anoikis-related genes (ARGs), their prognostic value and the relationship between these genes and the tumor microenvironment is of significant importance for a deeper understanding of GBM pathogenesis and the development of novel therapeutic strategies.

Non-negative matrix factorization (NMF) can reveal latent patterns of gene expression in single cells. By performing NMF decomposition on gene expression matrices, a set of gene expression patterns and their corresponding cell weights can be obtained [14]. This helps to identify potential cell subtypes and expression patterns, finely delineate immune cells in GBM, particularly TAMs, and clarify the unique gene signatures associated with each NMF cluster. This study uncovers the profound impact of anoikis-mediated changes in the tumor microenvironment (TME) at the single-cell level, finding that BRMS1 promotes M2 polarization in microglia and activates the PI3K/AKT signaling pathway through SPP1/CD44-mediated cell interactions, inhibiting GBM cell apoptosis and promoting proliferation, migration, and invasion, leading to adverse effects on the prognosis and immune therapy response of GBM patients.

## Materials and methods

### Sources of raw data

Single-cell RNA sequencing (scRNA-seq) data from eight GBM samples were obtained from TISCH2 (http://tisch.comp-genomics.org/) under the accession number GSE131928 [15]. All cells were annotated and preliminarily organized based on the cell types and dimensionality reduction data provided. Additionally, gene expression profiling data and associated clinical information from 168 glioblastoma samples were downloaded from The Cancer Genome Atlas Program (TCGA) via the UCSC Xena website (https://xena.ucsc.edu/). For validation purposes, gene expression profiling data from 374 GBM patients were sourced from the Chinese Glioma Genome Atlas (CGGA) data portal (http://www.cgga.org.cn/). In this study, gene expression levels were primarily estimated as log2 (TPM + 1). Spatial transcriptome data for primary GBM from the 10 × Visium platform were downloaded from GSE194329 [16]. A total of 35 anoikis-related genes (ARGs) were outputted from the Molecular Signatures Database (MSigDB, https://www.gsea-msigdb.org/gsea/msigdb) (Supplementary Table).

### Processing of single-cell sequencing data and spatial transcriptome sequencing data

The "Seurat" and "SingleR" R packages were utilized to process and analyze single-cell RNA sequencing (scRNA-seq) data [17]. After executing quality control (QC) and normalization procedures, the data underwent further normalization through the "NormalizeData" function. This processed data was then transformed into Seurat objects, from which the 2000 most variable genes were pinpointed using the "FindVariableFeatures" function. Principal component analysis (PCA) was subsequently conducted on these Seurat objects with the "ScaleData" and "RunPCA" functions to ascertain the most effective number of principal components (PCs). JackStraw analysis was applied to pinpoint the PCs that were significant, and these were chosen based on their contribution to variance for the purpose of cell clustering. The integration of data was clustered using the "FindNeighbors" and "FindClusters" functions. To manage and visualize high-dimensional data, uniform manifold approximation and projection (UMAP), a prevalent technique, was employed to group and dimensionally reduce the scRNA-seq data. The "FindAllMarkers" and "FindMarkers" functions from the "scran" R package facilitated the execution of Wilcoxon tests to identify genes that were specifically expressed within each cell cluster. The expression of these specific genes was then illustrated using the "featureplot" function. In conclusion, the primary cell types were identified and depicted using classifications from the TISCH2 official website and data from UMAP.

Spatial transcriptome data analysis was conducted within the R environment using the "Seurat" package. The data underwent a process of scaling and centering through the "SCTransform" function, which ensured uniformity in the mean and variance of the dataset, followed by a reduction in the data's dimensionality through the application of "RunPCA". In the context of unsupervised clustering, the "FindNeighbors" and "FindClusters" functions were executed using their default settings, incorporating the top 30 principal components deemed most critical for the analysis. Visualization of the data to reveal subpopulations and individual genes was achieved with the "SpatialFeaturePlot" function. "Scanpy" is recognized as a comprehensive Python-based toolkit designed for the analysis of single-cell RNA sequencing data. It encompasses a range of features such as data preprocessing, data visualization, clustering algorithms, and differential gene expression analysis, all of which facilitate a detailed examination of gene expression patterns at the single-cell level. "stLearn" is a Python library based on "Scanpy" that focuses on the analysis of spatial transcriptomics data. It integrates gene expression and image information to understand the relationship between cell position in tissue and gene expression. In summary, "Scanpy" is used for single-cell RNA sequencing data, while "stLearn" extends its capabilities for the analysis of spatial transcriptome data, helping researchers delve deeper into the spatial distribution of cells and gene expression within tissues. "SPOTlight" is an R package for spatial transcriptome deconvolution, designed to address single-cell expression data at spatial resolution [18]. The package provides powerful deconvolution tools that, using techniques such as non-negative matrix factorization, can decompose mixed signals of cell types into individual cell contributions, enabling spatial annotation and visualization.

### Identification of marker genes in different cell clusters by non-negative matrix factorization

To reduce data redundancy and noise and to better observe the impact of anoikis-mediated regulatory factor expression on TME cell types in gliomas, we further refined the clustering analysis based on the previously annotated cells using the "NMF" R package, thereby generating NMF cell clusters. Subsequently, we utilized the "FindAllMarkers" function to calculate the gene signature for each NMF cell cluster. The threshold for marker gene selection included a logfc. threshold > 1 and an adjusted *p*-value < 0.05. Furthermore, we named the NMF cell clusters according to the following rules. NMF cell clusters with positive ARG expression, characterized by an adjusted p-value of less than 0.05 and a logfc.threshold greater than 1, were annotated as corresponding gene signature cell clusters. Conversely, if the gene signature of the NMF cell cluster met the adjusted p-value and logfc.threshold criteria but these genes did not include any ARGs, the cluster was defined as a non-anoikis cluster. This decision-making process strictly followed the methodology of previous studies, ensuring the accuracy and reliability of the results [19].

### Pseudotime trajectory and cell–cell communication analysis

We conducted trajectory analysis using Monocle 2 [20] to explore the expression patterns of ARGs within TME cells in our scRNA-seq data. Initially, the "newCellDataSet" function was applied to construct the mycds object, and highly variable genes were identified through careful filtering. The "reduceDimension" function was utilized for dimensionality reduction, and the "DDRTree" method was applied for descending order sorting of the mycds object. Furthermore, cells were positioned onto the Pseudotime trajectory using the "orderCells" function with default parameters. Finally, a heatmap was visualized for the pseudotime trajectory analysis.

“CellChat” is an R package specifically designed for scRNA-seq and spatial transcriptomics data, aiming to infer, analyze, and visualize cellular communication networks [21]. By providing tools for the inference and visualization of interactions, users can gain insights into the interplay between different cell types, thereby revealing patterns and dynamics of cellular communication within biological systems. The acquired data is intuitively represented through various charts. In this study, we investigated the cell-to-cell communication networks between the target NMF cell clusters and other NMF cell clusters.

### SCENIC analysis and metabolism analysis

SCENEIC is an R package designed for the inference of gene regulatory networks and cell type clustering from single-cell RNA-sequencing data [22]. By integrating single-cell expression data, SCENEIC employs gene expression in conjunction with Gradient Boosting-based Gene Regulatory Network (GRNBoost). The process involves identifying potential targets of transcription factors (TFs), constructing gene regulatory networks, and performing cell-type clustering. Utilizing the RcisTarget database (https://resources.aertslab.org/cistarget/), which contains a gene-motif ranking (hg19-TSS-centric-10 kb), it detects transcription start sites (TSS) and gene regulatory networks in scRNA-seq data within GBM. Overall, the package achieves gene co-expression network inference based on GENIE3 and identifies transcription factor targets through RcisTarget. "scMetabolism" is another R package for assessing metabolic activity scores at the single-cell resolution. It offers user-friendly tools that allow researchers to study and quantify cellular metabolic activity at the single-cell level. Based on conventional single-cell matrix files, it employs the VISION algorithm to score each cell, providing insights into the metabolic state of individual cells and aiding in a deeper understanding of metabolic heterogeneity among cells [23].

### Survival analysis and immunotherapy prediction of NMF cell clusters

To investigate the impact of anoikis-related characteristics on the prognosis of glioblastoma, after obtaining the gene signatures of different NMF cell clusters, we utilized the R package "GSVA" to calculate the feature scores of NMF cell clusters in TCGA and CGGA cohorts. Based on the results obtained, we conducted univariate Cox regression analysis and Kaplan–Meier survival analysis to assess the influence of the proportion of different NMF cell clusters on the survival of GBM patients. Different NMF cell cluster score groups were defined according to the optimal cutoff values. To explore the effect of anoikis-related characteristics on the immune therapy response in GBM patients, we performed an analysis of ICB. The Tumor Immune Dysfunction and Exclusion (TIDE) online platform (http://tide.dfci.harvard.edu/) was used for the prediction of immunotherapy in GBM samples and to investigate the scores of each NMF cell cluster in the responder and non-responder groups.

### Cell culture and transfection

The human glioma cell lines U251 and U118, as well as the human microglia cell line HMC3, were all sourced from the Shanghai Cell Bank of the Chinese Academy of Sciences and cultured in Dulbecco's Modified Eagle Medium (DMEM; Gibco, Carlsbad, CA) supplemented with 10% Fetal Bovine Serum (FBS). These cell lines have been authenticated using Short Tandem Repeat (STR) profiling by GeneWiz, Inc. and confirmed to be free from mycoplasma contamination.

The lentiviral vector for BRMS1 overexpression was constructed by Genechem (GXDL0168387). The full-length cDNA of human BRMS1 and its sequence deletion mutants were amplified by PCR and verified by DNA sequencing. These sequences were cloned into the lentiviral vector GV348 with a Flag tag.

GBM cells, either untreated or treated with lentivirus, were co-cultured with HMC3 microglia cells in 6-well plates (Nunc™ Polycarbonate Cell Culture Treatises with 0.4 μm pore size) for 72 h. This setup allowed for the exchange of culture medium components while preventing cell migration.

### Quantitative real-time PCR, western blot analysis, and enzyme-linked immunosorbent assay (ELISA)

Total RNA was extracted from cells using TRIzol reagent (Invitrogen) according to the manufacturer's protocol. Subsequently, quantitative polymerase chain reaction (qPCR) analysis was conducted on the LightCycler 480 II instrument (Roche Applied Science) using Real SYBR Mixture (CoWin Bioscience, China). Primer sequences for BRMS1 and GAPDH genes were designed as follows: BRMS1 forward primer 5'-TGCAGCGGAGCCTCAAG-3', reverse primer 5'-TCACATCCAGACAGAACGCCT-3'; GAPDH forward primer 5'-GGAGCGAGATCCCTCCAAAAT-3'. GAPDH gene expression levels were used for normalization as an internal control. Relative changes in target gene expression were analyzed using the comparative CT (ΔΔCT) method.

Total protein was extracted from cells using RIPA lysis buffer (Sigma Aldrich) containing a cocktail of protease inhibitors. Protein concentrations were determined using the BCA Protein Assay Kit (Sigma Aldrich). Proteins were separated by standard 10% SDS-PAGE and transferred onto PVDF membranes (Millipore). Membranes were incubated overnight at 4 °C with primary antibodies (Proteintech) and then with secondary antibodies at room temperature for 1 h after washing with TBST solution. Protein bands were visualized using an ECL kit (Millipore). The primary antibodies used in this experiment were: GAPDH (dilution 1:3000, Proteintech, Wuhan, product number 60004–1-Ig); BRMS1 (dilution 1:1000, Proteintech, Wuhan, product number 16096–1-AP); CD204 (dilution 1:1000, Abcam, Shanghai, product number ab123946); CD209 (dilution 1:800, Proteintech, Wuhan, product number 25404–1-AP); TGF-β1 (dilution 1:1000, Abcam, Shanghai, product number ab315254); SPP1 (dilution 1:1000, Proteintech, Wuhan, product number 80912–4-RR); CD44 (dilution 1:1000, Proteintech, Wuhan, product number 15675–1-AP); Phosphorylated AKT (*p*-AKT, dilution 1:1000, Proteintech, Wuhan, product number 66444–1-Ig); PI3K (dilution 1:800, Abcam, Shanghai, product number ab283852); AKT (dilution 1:800, Abcam, Shanghai, product number ab283852); Cleaved-Caspase3 (dilution 1:800, Abcam, Shanghai, product number ab214430); Bax (dilution 1:800, Abcam, Shanghai, product number ab53154); Bcl-2 (dilution 1:1000, Abcam, Shanghai, product number ab194583).

Enzyme-linked immunosorbent assay (ELISA) was performed using the supernatant from HMC3 microglia cell cultures to detect the expression levels of CXCL2, IL-10, and TGF-β1 using respective ELISA kits (product numbers ab184862, ab100549, and ab100647, all from Abcam, Cambridge, UK). The ELISA procedures were carried out precisely according to the manufacturer's instructions to ensure reliable results. These assays allowed for the quantitative analysis of the concentrations of these key cytokines in cell culture supernatants, thereby assessing the role and function of microglia in various biological processes.

### Cell counting kit-8 assay, colony-forming assay, EdU assay, in vitro migration, invasion assays, and apoptosis experiments

To measure cell proliferation, we utilized the Cell Counting Kit-8 (CCK-8) (Dojindo, CK18, Japan). Control and transfected cells were seeded at an initial density of 1000 cells per well in 96-well plates (Corning, 3599, America), with 10 μl of CCK-8 reagent added per well; cell viability was analyzed using a microplate reader (Thermo, Multiskan-Spectrum, America) 24 h later, designated as day 0. Subsequently, cell viability was measured every 24 h. From day 1 to day 5, cell growth was represented by fold change and plotted as a line graph.

Control and treated cells were plated at a density of 1000 cells per well in 6-well plates (Corning, America) and incubated continuously for two weeks until visible colonies were observed. Colonies were fixed with 4% paraformaldehyde (Biosharp, BL538A, China) and stained with 0.1% crystal violet (BBI, E607309, China) for 15 min at room temperature. After photography, the total number of colonies was counted using ImageJ software.

Cell proliferation was assessed using the EdU assay. Cells were cultured for 1 h at 37 °C with EdU reagent (Invitrogen, C10639, Canada) at a concentration of 10 μM. The culture medium was then discarded, and cells were fixed with 4% paraformaldehyde for 30 min at room temperature, followed by permeabilization with 1% Triton X-100 (Biosharp, BS084, China) for 20 min. Cells were stained according to the manufacturer's protocol and imaged using a fluorescence microscope (Leica Microsystems GmbH, Mannheim, Germany). Image compositing and cell counting were performed using Fiji:ImageJ. The growth and proliferation rate were represented by the ratio of EdU-positive cells to DAPI-positive cells.

Cell invasion assays were conducted using Transwell chambers with 8 μm pore size (Millipore, Billerica, MA, USA) and 15 mg/mL concentration of Matrigel (BioScience, Bedford, MA, USA). Two hundred microliters of culture medium containing 0.1% fetal bovine serum were added to the lower chamber, and 10^5 cells were cultured in 600μL of medium with 10% fetal bovine serum as a chemoattractant in the upper chamber. After a 48-h incubation, non-invading cells in the upper chamber were removed, and cells that had invaded to the lower chamber were fixed and stained with crystal violet. The number of invading cells was quantified by dissolving the stained cells in 3% glacial acetic acid and measuring the absorbance of the solution at 450 nm.

Apoptosis analysis was performed using the Annexin V-FITC/7-AAD apoptosis detection kit (Sigma-Aldrich, St. Louis, MO, USA) according to the manufacturer's instructions. Samples were analyzed using a flow cytometer (Cytoflex, Beckman, CA, USA). The determination of apoptosis rates included both early and late apoptotic cells.

### Statistical analysis

All analyses were performed using R version 4.1.3 or Python version 3.9. To compare patient survival among different subgroups within each dataset, Kaplan–Meier survival analysis and log-rank tests were employed. The significance of differences between groups was assessed using Kruskal–Wallis and Wilcoxon tests. Spearman's correlation analysis was used to examine the correlation coefficients. A *P*-value of less than 0.05 was considered statistically significant in all analyses.

## Results

### Cellular characteristics in the tumor microenvironment of glioblastoma and distribution of anoikis-related genes

Neftel et al. previously classified glioblastoma cells into four subtypes using scRNA-seq, including neural progenitor-like cells (NPC-like), oligodendrocyte progenitor-like cells (OPC-like), astrocyte-like cells (AC-like), and mesenchymal-like cells (MES-like). Importantly, extensive research has revealed a transition from OPC-like/NPC-like malignant cells to MES-like malignant cells, indicating a high degree of dynamic plasticity in GBM cells [15]. Hara et al. demonstrated that the expression of a macrophage mesenchymal program induced the transition of glioblastoma cells to a mesenchymal-like (MES-like) state, which is also associated with increased cytotoxicity of T cells [24]. Therefore, a thorough understanding of the extensive changes in the immunological microenvironment of macrophages and T cells has potential therapeutic significance for GBM.

**Fig. 1:**
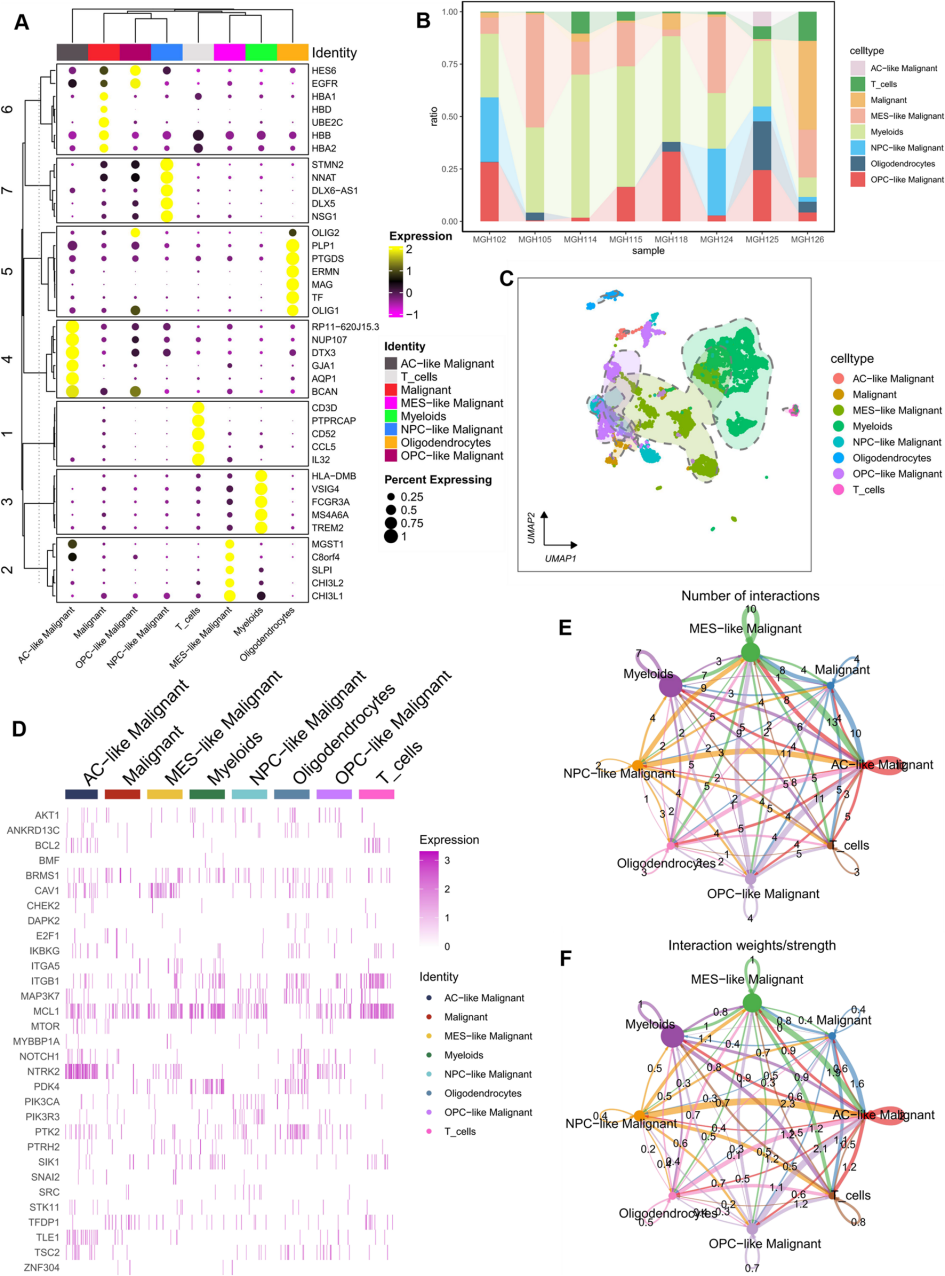
Overview of ARGs in GBM scRNA-seq data. (**A**) Bubble chart distribution of top marker genes in each cell type. (**B**) Proportion of each cell type among eight GBM samples. (**C**) Single-cell UMAP diagrams of each cell type are provided by the TISCH2 website. (**D**) Heatmap of ARG expression in each cell type. (**E**, **F**) Analysis of cell–cell communication intensity and quantity between cell types through Cellchat

Initially, we downloaded GBM scRNA-seq data (GSE131928) from the TISCH2 website and annotated all cells based on the provided cell types. A total of 8 cell types, including OPC-like malignant cells, AC-like malignant cells, NPC-like malignant cells, MES-like malignant cells, malignant cells, oligodendrocytes, T cells, and myeloid cells, were annotated. Figure 1B shows the proportion of different cell types in 8 samples, with myeloid cells being the main component in all samples. UMAP was used for dimensionality reduction clustering to display the distribution of different cell types and tumor heterogeneity (Fig. 1C). The most prominent marker genes for each cell type were presented in the form of a bubble chart (Fig. 1A). CellChat builds cell–cell communication probabilities based on a matrix of single-cell gene expression quantities by integrating a priori knowledge of the interactions between gene expression and signalling ligands and receptors, which in turn makes predictions about intercellular communication networks. Therefore, we also examined the number and strength of intercellular communication signals between these eight cell types, with the number and strength labelled with specific numbers (Fig. 1E, F). For example, the highest strength and number of communications were found between MES-like malignant cells and OPC-like malignant cells. The expression patterns of 35 ARGs in different cell types are shown in Fig. 1D, with MCL1 being relatively more highly expressed in all cells, and NTRK2 being most highly expressed in AC-like malignant cells. These cellular distribution characteristics provide valuable insights into the potential functions of anoikis.

### Characteristics of anoikis-mediated CD8 + T cells in glioblastoma

By analyzing the marker genes of T lymphocytes, we identified CD4 + T cells (marker genes: CD3E, CD4, IL7R, IL2RA, and FOXP3), CD8 + T cells (marker genes: CD3E, CD8A, CCL5, and KLRB1), and NK cells (marker genes: NCR1, KLRB1, and NKG7) (Supplementary Fig. 1B). Pseudotime trajectory analysis of CD8 + T cell differentiation showed that ARGs such as TLE1, MTOR, PIK3R3, ITGB1, and MAP3K7 were progressively upregulated, while E2F1, NTRK2, DAPK2, BRMS1, and NOTCH1 were progressively downregulated (Supplementary Fig. 1A). NMF annotation of CD8 + T cells resulted in 3 clusters, including ITGB1 + CD8 + T cells-C1, Non-Anoikis-CD8 + T cells-C2, and BCL2 + CD8 + T cells-C3 (Supplementary Fig. 1C). Intercellular communication analysis revealed that all 3 NMF cell clusters had significant cellular communication links only with MES-like malignant cells, with no apparent cellular interactions with other types of malignant cells (Supplementary Fig. 1D, E). MES-like malignant cells exhibited various signal output patterns, including MIF, PTN, SPP1, CXCL, and ANGPTL, while signal output patterns from neighboring cells mainly included SPP1 and ANGPTL (Supplementary Fig. 1F). Additionally, SCENIC analysis was performed on anoikis-related NMF cell clusters, presenting the activity levels of 9 transcription factors in the form of UMAP (Supplementary Fig. 1G). Notably, in the BCL2 + CD8 + T cells-C3 group, the expression activity of JUND, JUNB, FOS, YBX1, and FOXB was the highest. In the ITGB1 + CD8 + T cells-C1 group, SF1, IRF1, and BHLHE40 had relatively higher activity (Supplementary Fig. 1H). Notably, there were differences in the expression levels of exhaustion and cytotoxic T cell characteristic genes in different CD8 + T cell clusters. Overall, cluster C1 had a higher exhaustion score, while cluster C2 had a higher cytotoxicity score (Supplementary Fig. 1J). Finally, we also studied the expression of immune suppressive signaling molecules in different NMF cell clusters, with PD1 being highly expressed in the C2 subgroup with T cell cytotoxicity functions (Supplementary Fig. 1I).

### Characteristics of anoikis-mediated microglia in glioblastoma

Since in vivo glioma-associated myeloid cell subtypes do not directly correspond to in vitro-defined M0, M1, or M2-like macrophages, we defined microglia based on the expression levels of CX3CR1, TMEM119, ADORA3, BHLHE41, and BIN1 [25, 26], mast cells based on MS4A2, CPA3, KIT, and TPSAB1 [27], and other tumor-associated monocytes/macrophages based on HLA-DRB5, S100A11, and MS4A6A [25]. A recent study further divided microglia into i-Mic, h-Mic, AP-Mic, and a-Mic [28]. i-Mic mainly expressed activation markers of microglia, such as CCL3/MIP-a, CCL4/MIP-β, CCL3L3, CCL4L2, CD83, TNF, IL1B, and NFKBIZ. h-Mic highly expressed CST3, a marker of homeostatic microglia. AP-Mic had characteristics of both microglia and macrophage markers, while highly expressing CX3CR1, CD86, IFNGR1, TGFB1, and B2M. Finally, a-Mic was separated from i-Mic and h-Mic based on different expression levels of SPRY1, PYRY13, and microglial activation markers. Interestingly, this study showed that i-Mic, h-Mic, and a-Mic were all associated with better prognosis in glioma patients, while AP-Mic represented poorer survival rates and exhibited metabolic characteristics of immunosuppressive macrophages. Therefore, our study focused on AP-Mic as the main research object.

**Fig. 2:**
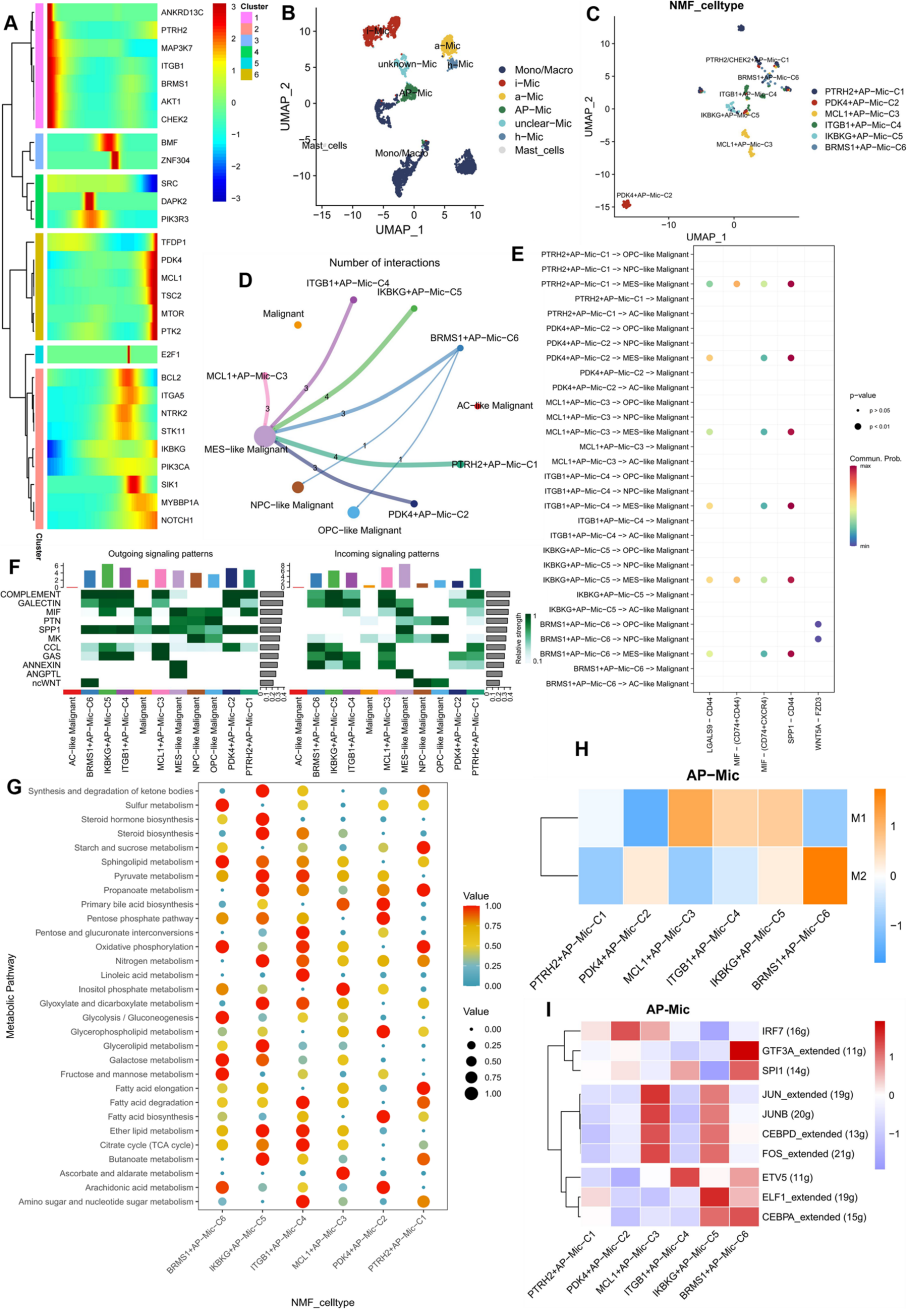
The role of anoikis-mediated microglia in the TME. (**A**) Heatmap of trajectory analysis of ARGs in myeloid cells. (**B**) Isolation of i-Mic, h-Mic, AP-Mic, a-Mic, and other macrophages from myeloid cells through specific marker genes, with distribution characteristics displayed by UMAP. (**C**) Further downscaled clustering of AP-Mic using NMF and cell cluster annotation, with cell distribution characteristics shown by UMAP. (**D**) Communication quantity between AP-Mic NMF clusters and various malignant cells. (**E**) Receptor-ligand pairs between AP-Mic NMF clusters and various malignant cells. (**F**) Heatmap showing incoming and outgoing interactions between AP-Mic NMF clusters and various malignant cells. (**G**) Heatmap displaying significantly different metabolic signaling pathways in AP-Mic NMF clusters. (**H**) Calculation of polarization scores for six AP-Mic NMF clusters based on marker genes of M1 and M2 macrophages. (**I**) Heatmap showing significantly different TF activities among AP-Mic NMF clusters using AUCell's average score

UMAP was used for dimensionality reduction clustering to display the distribution of monocytes/macrophages, mast cells, i-Mic, h-Mic, AP-Mic, a-Mic, and undefined microglia (Fig. 2B). To study the dynamics of AP-Mic differentiation, we performed pseudotime trajectory analysis and found that ARGs such as ANKRD13C, PTRH2, MAP3K7, ITGB1, BRMS1, AKT1, and CHEK2 were mainly expressed in the early stages of AP-Mic differentiation, while TFDP1, PDK4, AQP5, MCL1, TSC2, MTOR, and PTK2 were concentrated in the later stages of cell development (Fig. 2A). Subsequently, NMF clustering annotation of AP-Mic identified 6 different NMF cell clusters (PTRH2 + AP − Mic − C1, PDK4 + AP − Mic − C2, MCL1 + AP − Mic − C3, ITGB1 + AP − Mic − C4, IKBKG + AP − Mic − C5, BRMS1 + AP − Mic − C6) (Fig. 2C). Communication analysis between the 6 NMF cell clusters and various malignant cells revealed their complex intercellular interactions. Interestingly, all NMF cell clusters showed significant links with MES-like malignant cells, with only the BRMS1 + AP − Mic − C6 group having cellular communication with NPC-like/OPC-like malignant cells (Fig. 2D). Notably, MES-like malignant cells mainly exhibited signal output patterns including ANNEXIN, ANGPTL, and MIF, as well as signal input patterns including SPP1, ANGPTL, and MIF (Fig. 2F). Figure 2E shows potential receptor-ligand pairs between the 6 NMF cell clusters and various malignant cells. Using macrophage polarization-related marker genes to detect the correlation between the 6 NMF cell clusters and M1/M2 macrophage subtypes, it was found that the BRMS1 + AP − Mic − C6 group had a higher M2 score (Fig. 2H). Additionally, we studied the biochemistry within the 6 anoikis-related NMF cell clusters. Interestingly, the BRMS1 + AP − Mic − C6 group significantly enriched the pathway of oxidative phosphorylation (Fig. 2G). Finally, the heatmap presented the transcription factor activity of the 6 NMF cell clusters (Fig. 2I). In the BRMS1 + AP − Mic − C6 group, the activity of GTF3A, SPI1, and CEBPA was relatively higher. Combined with the above analysis, we preliminarily speculate that BRMS1 seems to further promote the M2 phenotype polarization in microglia with certain immunosuppressive phenotypes.

### Anoikis-mediated TME can accurately predict prognosis and immunotherapy response

In the TCGA cohort, using ssGSEA, the tumor group showed higher anoikis enrichment scores (Supplementary Fig. 2A). To assess the impact of anoikis-regulated immune cells on the prognosis and immunotherapy efficacy of GBM, we collected signature genes from each NMF cell cluster and used them to score samples in bulk RNA-seq (TCGA cohort and CGGA cohort). Using ssGSEA, we calculated the enrichment scores of different NMF cell clusters in each sample and studied the prognostic correlation of anoikis-regulated TME cells in GBM. Comparing NMF cell cluster scores between normal and tumor groups in TCGA, both CD8 + T cells and AP-microglia showed higher proportions in tumor samples (Supplementary Fig. 2B). Supplementary Fig. 2C shows the complex cellular communication links between the 2 defined CD8 + T cell clusters and 6 AP-microglia cell clusters. Subsequently, using the clinical information of each sample for univariate Cox regression analysis, we calculated the odds ratios (ORs) for each NMF cell group. The analysis showed that most NMF cell clusters in GBM were associated with poorer prognosis (Supplementary Fig. 2F). Based on the ssGSEA scores, we equally divided the GBM patients into high and low expression groups of BRMS1 + AP-Mic-C6. kaplan–Meier survival analysis found that high expression of BRMS1 + AP-Mic-C6 represented a poorer survival outcome in both TCGA and CGGA datasets (Supplementary Fig. 2D, E).

Using TIDE to perform ICB analysis on tumor samples from TCGA and CGGA, predictions were made for each GBM sample's response to immunotherapy, and then the enrichment abundance of different NMF cell clusters in ICB responder and non-responder groups was compared. In the TCGA cohort, low expression levels of PTRH2 + AP − Mic − C1 and high expression levels of PDK4 + AP − Mic − C2 showed effective immune responses (Supplementary Fig. 2G). In the CGGA cohort, in addition to PDK4 + AP − Mic − C2, a lower abundance of other NMF cell clusters favored immunotherapy for GBM (Supplementary Fig. 2H). Subsequently, using ICB response and no ICB response as binary variables, logistic regression analysis was performed on each sample's NMF cell clusters. The results showed that in the CGGA dataset, the expression of BRMS1 + AP − Mic − C6 was significantly detrimental to GBM immunotherapy, while in the TCGA cohort, the results were not significant (Supplementary Fig. 2I). In summary, microglia with positive BRMS1 expression are likely to lead to poor prognosis and poor immunotherapy response in GBM patients.

### Cellular communication between BRMS1 + microglia and malignant cells in spatial transcriptomics

**Fig. 3:**
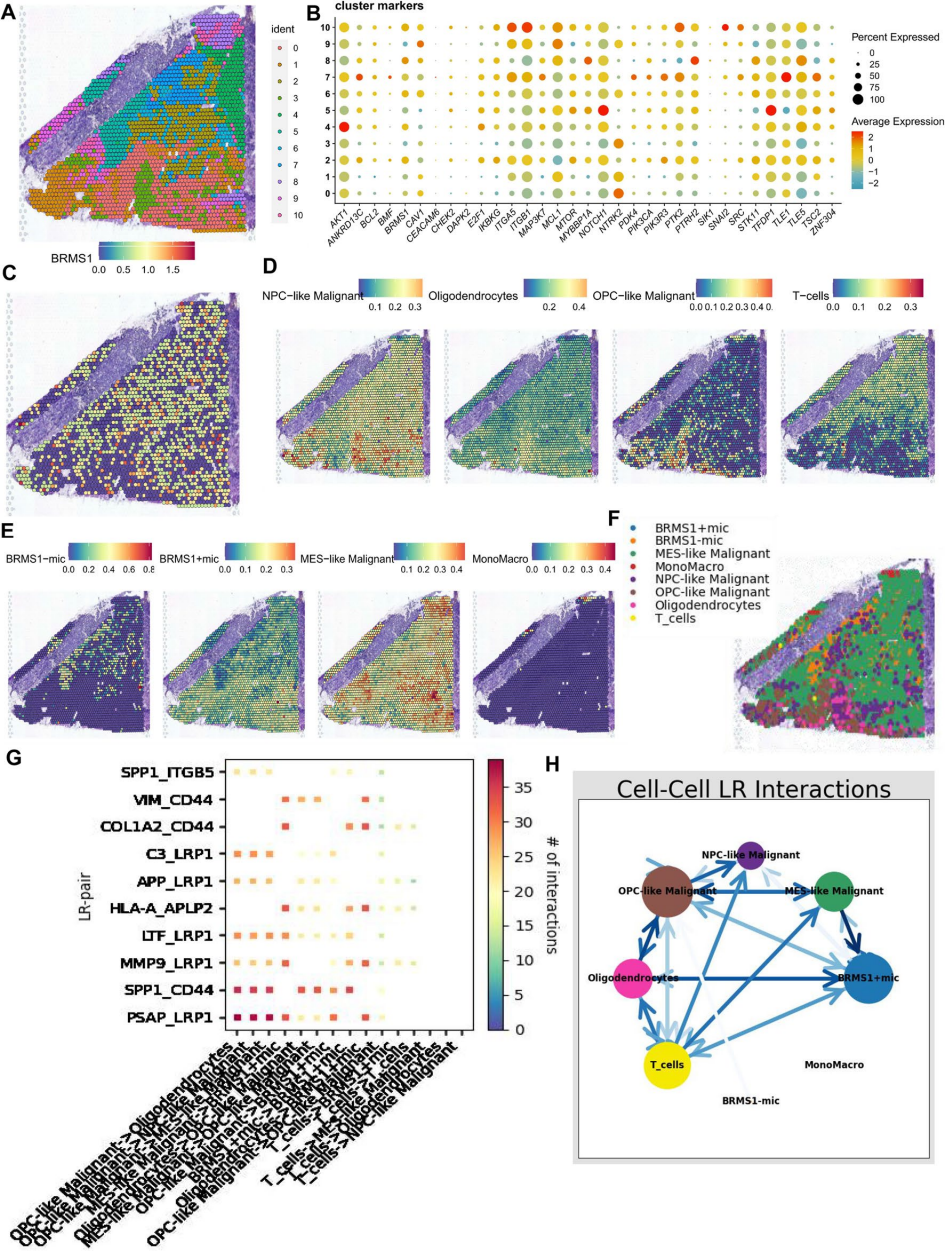
Cell communication between BRMS1 + microglia and malignant cells in spatial transcriptomics. (**A**) Spatial map showing 11 clusters identified by stRNA-seq. (**B**) Bubble chart presenting the expression of ARGs in different clusters. Red represents high expression, and blue represents low expression. (**C**) Spatial map showing the expression of BRMS1 in GBM. (**D**, **E**) Identification of cell types and proportions in each spot through deconvolution methods, with spatial maps showing the proportion and expression of different cell types, especially BRMS1 + and BRMS1- microglia. (**F**, **G**) Heatmaps and network diagrams calculate the communication intensity between different cell types based on the stlearn method

Spatial transcriptome sequencing data from a GBM patient were acquired from the dataset GSE194329. Once the ribosomal and mitochondrial genes were excluded, the SCTransform approach was implemented to adjust for sequencing depth and to carry out a suite of standardization steps. Following the application of dimensionality reduction techniques and subsequent clustering, a total of 11 distinct cellular subgroups were identified across the spatial landscape (Fig. 3A). Figure 3B shows the expression of 35 ARGs in these 11 subgroups. BRMS1 had relatively abundant gene expression values in the GBM section (Fig. 3C). Based on whether BRMS1 is expressed in microglia, we divided scRNA-seq microglia into positive and negative expression groups. To understand the spatial interactions of these cells, we combined single-cell transcriptome data with spatial data using SPOTlight to identify cell types and proportions in each spot (Fig. 3D, E). Spots were defined based on the highest proportion of cell types in each spot (Fig. 3F). Finally, we inferred the spatial connections between BRMS1 + microglia and malignant cells in complex GBM tissue. Interestingly, BRMS1 + microglia had stronger receptor-ligand communication links with other malignant cells, especially MES-like malignant cells, while BRMS1- microglia and other macrophages hardly participated in cellular interactions with malignant cells (Fig. 3G, H). Moreover, both stRNA-seq and scRNA-seq indicated that BRMS1 + microglia may promote the proliferation and invasion of malignant glioma cells through SPP1/CD44-mediated intercellular interactions.

### BRMS1 promotes microglial M2 polarization and activates the PI3K/AKT signaling pathway in GBM cells

Initially, to mimic the glioma microenvironment, we utilized a co-culture system of HMC3 microglia and GBM cells for our study. Furthermore, to examine whether BRMS1 could influence the M2 phenotype polarization of microglia, we transfected HMC3 cells with a BRMS1-overexpressing lentivirus. Twenty-four hours post-transfection, both transfected and non-transfected HMC3 cells were co-cultured with U118/U251 cells in a six-well plate setup for 72 h.

**Fig. 4:**
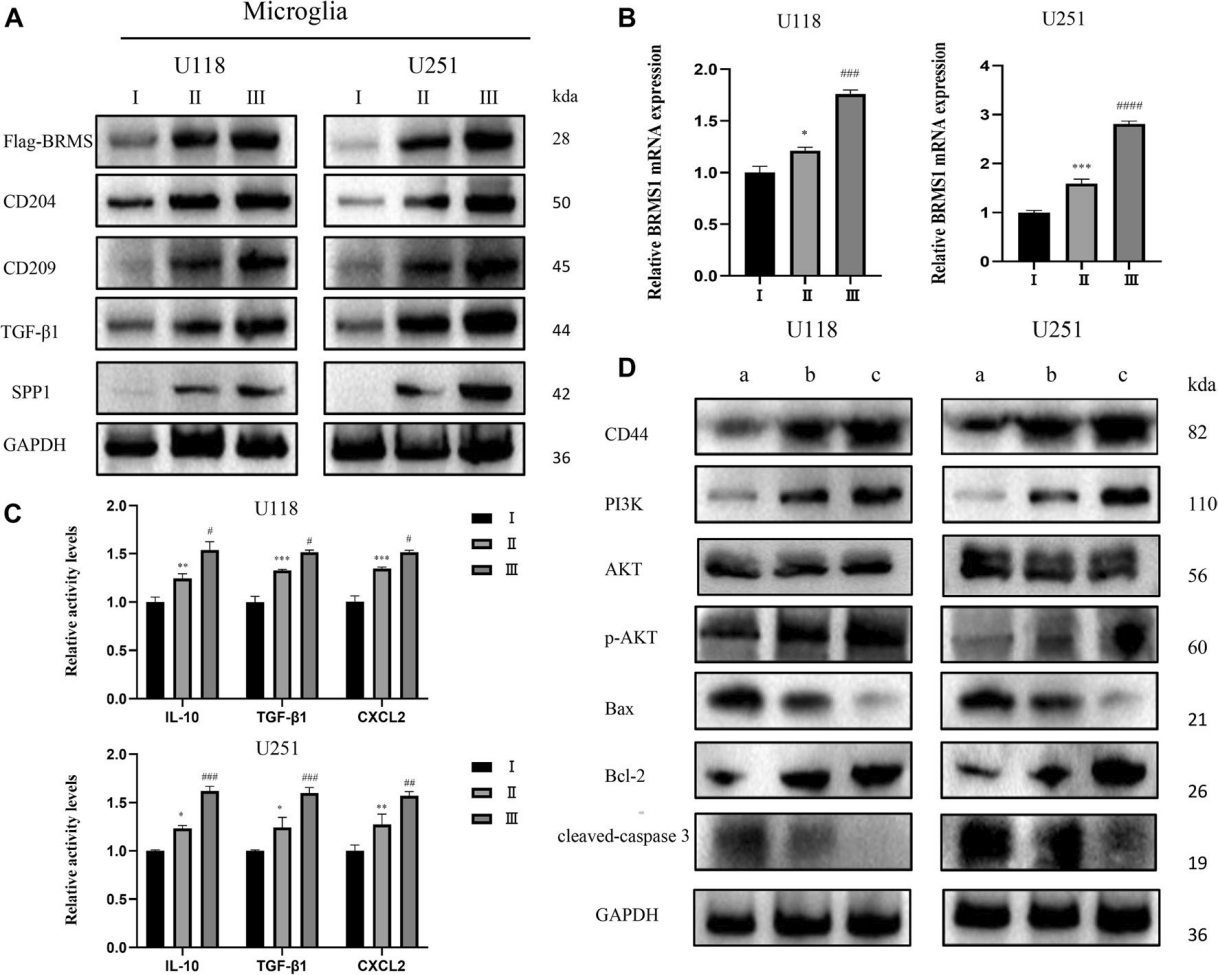
BRMS1 promotes M2 polarization of microglia and activates the PI3K/AKT signaling pathway in GBM cells. (**A**) Western blot analysis was used to measure the protein levels of CD209, CD204, TGF-β1, BRMS1, and SPP1 in HMC3 cells after co-culture with U118/U251 cells. (**B**) RT-qPCR was performed to detect the RNA expression of BRMS1 in HMC3 cells from different groups. (**C**) ELISA was employed to assess the activity levels of CXCL2, IL-10, and TGF-β1 in the supernatant of HMC3 cell cultures. (**D**) Western blot analysis was conducted to evaluate the expression levels of CD44, PI3K, p-AKT, Bax, Bcl2, and cleaved-caspase 3 in U118/U251 cells following co-culture with HMC3 cells. Group I: Untreated HMC3 cells; Group II: HMC3 cells co-cultured with U118/U251 cells; Group III: HMC3 cells transfected with Flag-BRMS1 and co-cultured with U118/U251 cells. a: Untreated U118/U251 cells; b: U118/U251 cells co-cultured with HMC3 cells; c: U118/U251 cells co-cultured with Flag-BRMS1-transfected HMC3 cells. All data are represented as the mean ± SD of three independent experiments. * *p* < 0.05, ** *p* < 0.01, *** *p* < 0.001 for comparisons between Group II and Group I. # *p* < 0.05, ## *p* < 0.01, ### *p* < 0.001, #### *p* < 0.0001 for comparisons between Group III and Group II

The results indicated that compared to the blank control group, the expression levels of M2 macrophage-specific markers (CD209, CD204, TGF-β1) in the lysates of HMC3 microglia and the supernatants (CXCL2, IL-10, TGF-β1) significantly increased after co-culture. This suggests that the supernatant from glioma cell cultures can polarize microglia towards the M2 macrophage phenotype. Additionally, the protein expression levels of BRMS1 and SPP1 in microglia were also notably upregulated relative to the blank control group after co-culture. Interestingly, transfection with Flag-BRMS1 significantly upregulated the expression of these M2 markers and SPP1 compared to HMC3 cells after co-culture (Fig. 4A, B, C). These results indicate that the expression of BRMS1 is involved in the M2 polarization and the differentiation of the SPP1 + phenotype in microglia.

Our further experiments and bioinformatics studies suggest that the role of SPP1/CD44 mediated cell-to-cell interactions is likely a key factor in promoting the proliferation and invasion of GBM. Notably, CD44 binds to hyaluronic acid (HA), promoting the phosphorylation of its intracellular domain, which in turn further activates the PI3K/Akt signaling pathway [29]. Therefore, in the mechanism of glioma cell apoptosis, we examined the expression levels of PI3K/Akt signaling pathway proteins PI3K, AKT, and p-AKT, as well as downstream apoptosis-related proteins Bax, Bcl2, and cleaved-caspase 3. Corresponding to the previous co-culture method, HMC3 cells transfected and not transfected with lentivirus were co-cultured with U118/U251 cells in a six-well plate setup for 72 h. The results of immunoblotting showed that co-culture with HMC3 significantly increased the expression of CD44 in glioma cells, thereby activating the PI3K/AKT signaling pathway, leading to a significant downregulation of Bax and cleaved-caspase 3, and a significant upregulation of Bcl-2. The trend was further exacerbated in GBM cells co-cultured with HMC3 cells that overexpressed BRMS1 (Fig. 4D). In summary, our results show that the upregulation of BRMS1 in microglia may activate the PI3K/AKT signaling pathway in GBM through SPP1/CD44-mediated interactions, regulate the expression of apoptosis-related proteins Bax, Bcl2, and cleaved-caspase 3, thereby inhibiting the apoptosis of GBM cells.

### Upregulation of BRMS1 in microglia promotes glioma cell proliferation, migration, and invasion, and inhibits cell apoptosis

**Fig. 5:**
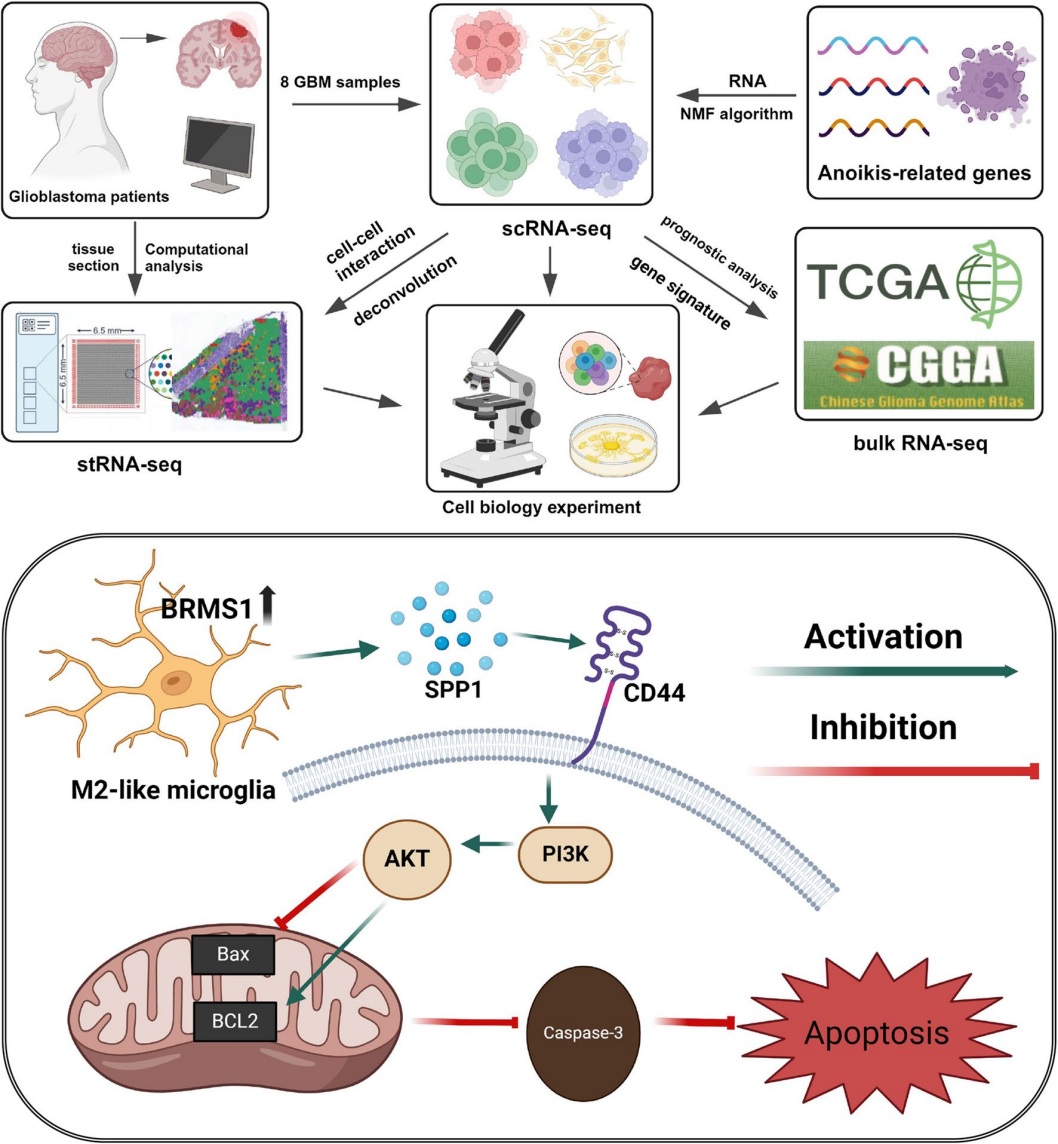
Summary of the bioinformatics research approach and experimental design principles of this study

Subsequently, to demonstrate that BRMS1 expressed in microglia plays a key role in the glioma microenvironment and can alter the proliferation and invasive capabilities of glioma cells, we conducted in vitro functional experiments. HMC3 cells, either transfected or not, were co-cultured with U118/U251 cells in a six-well plate setup for 72 h. The CCK-8 assay results and the EdU assay indicated that the co-culture environment and the overexpression of BRMS1 in HMC3 cells had a promoting effect on the proliferation of U118/U251 cells (Supplementary Fig. 3A, D). Similarly, the overexpression of BRMS1 in microglia enhanced the clonogenic ability of GBM cells (Supplementary Fig. 3C). Apoptosis in each group of cells was detected using flow cytometry, and we found that the overexpression of BRMS1 in microglia significantly inhibited apoptosis in GBM cells (Supplementary Fig. 3B). Transwell assays showed that the overexpression of BRMS1 in HMC3 cells significantly promoted the migration and invasion of GBM cells (Supplementary Fig. 3E, F). Finally, we summarized the bioinformatics design rationale and the specific cellular mechanisms throughout the study (Fig. 5).

## Discussion

Recent studies have revealed associations between the pathogenesis of glioblastoma and anoikis [30–32]. However, there is limited discussion on the potential role of anoikis-mediated immune cell regulation in tumorigenesis. This study is the first to comprehensively and deeply explore the mechanisms by which two main types of immune cells in the GBM tumor microenvironment, namely CD8 + T cells and microglia, are regulated by anoikis. Through in-depth analysis of single-cell RNA sequencing and spatial transcriptomics, we further identified the intricate cell–cell interactions between immune cell subtypes related to anoikis and tumor cells. This innovative approach provides researchers with valuable insights, helping to more fully understand the impact of different anoikis-mediated immune cell subtypes on the individual clinical outcomes of GBM patients.

In line with the 2021 glioma classification by the World Health Organization, GBM is categorized as a type with wild-type isocitrate dehydrogenase (IDH). GBM's varied cellular structure and histological presentation earn it the label of "pleomorphic," marking it as one of the most variably composed tumors [33]. The current therapeutic arsenal for GBM comprises surgical excision, radiation, and chemotherapeutic strategies, but the persistent challenge of tumor recurrence endures. A multitude of single-cell RNA sequencing investigations into gliomas have underscored the importance of tumor heterogeneity and the fluidity of cellular states as defining traits of these aggressive brain cancers. The inherent malleability of GBM introduces an additional dimension of complexity, permitting tumor cells to dynamically adjust throughout the stages of tumor initiation, progression, and evasion from therapy [34]. This adaptive capacity manifests across four principal states: mesenchymal-like (MES-like), neural progenitor cell-like (NPC-like), astrocyte-like (AC-like), and oligodendrocyte precursor cell-like (OPC-like). The characteristics of NPC, OPC, and AC-like states parallel those seen in neurodevelopmental pathways, whereas MES-like cells lack any counterpart in healthy brain tissue. Each GBM encompasses a variety of cell states that are not hierarchically compatible with one another. These transcriptomic profiles are loosely associated with the display of cell membrane antigens. In vivo, these marker-defined states possess varying degrees of tumorigenic potential and reflect the transcriptomic diversity of the original tumor [15]. The pronounced plasticity of GBM cells is inherently linked to their adaptive responses to the ever-changing microenvironment. Despite brain tumors' transcriptomic evolution and adaptations akin to those of the developing brain, they have often been isolated in research, overlooking the influence of the tumor microenvironment on cancer development. Within the GBM tumor microenvironment (TME), a consortium of stromal and immune-infiltrating cells collaborate to foster tumor growth and facilitate immune avoidance [35].

T cell exhaustion is a general term that refers to all states of functional impairment in antigen-specific CD8 + T lymphocytes. In recent years, researchers have primarily attributed the continuous stimulation by tumor antigens to the state of dysfunction in CD8 + T cells that are originally capable of recognizing and eliminating tumor cells. Our results are consistent with previous studies, where the expression of PD-L1 in GBM is associated with poor prognosis, thus GBM cells can regulate the expression of the PD-L1/PD-1 axis to evade immune surveillance and suppress the antitumor activity of CD8 + T cells [36]. In this study, compared to non-anoikis-mediated CD8 + T cells, more pronounced intercellular communication was observed between anoikis-mediated CD8 + T cells and MES-like tumor cells, especially in the ITGB1 + CD8 + T cell-C1 cluster. The integrin subunit β1 (ITGB1) is a transmembrane glycoprotein that can induce focal adhesion kinase (FAK) and AKT phosphorylation during cell growth and apoptosis processes [37, 38]. ITGB1 has been identified as an oncogene, and ITGB1 has been shown to promote the development of various cancers, including pancreatic cancer, gliomas, and cervical cancer [39–41]. However, the specific molecular mechanisms by which ITGB1 regulates T cells and mediates an immunosuppressive microenvironment require further investigation. Our study indicates a clear association between ITGB1 + CD8 + T cell clusters and the exhausted phenotype. Consistent with our findings, Yang et al. demonstrated through single-cell sequencing that ITGB1 can suppress the activation of immune T cells by upregulating macrophages and neutrophils, leading to immune escape of pancreatic cancer cells and reduced response rates to immune checkpoint therapy [42].

Increasing research suggests that targeting immunosuppressive macrophages may be key to improving the efficacy of immunotherapy, and bone marrow-derived cells can infiltrate malignant brain tumors and regulate tumor biology. However, the M1/M2 cell states determined in vitro do not represent the specific states of tumor-associated macrophages in the brain [43]. In addition to tumor-associated macrophages, the macrophage population in gliomas also includes microglia and myeloid-derived suppressor cells (MDSCs). Microglia are differentiated macrophages that become resident cells of brain tissue from immature macrophages during embryonic development of the brain [44]. Overall, the number of M2-type microglia is positively correlated with the grade of gliomas and can lead to an immunosuppressive microenvironment in gliomas. Microglia can secrete a variety of cytokines, growth factors, enzymes, and reactive oxygen species, directly or indirectly leading to angiogenesis, tumor proliferation, and tumor invasion in gliomas [45]. Meanwhile, cytokines secreted by gliomas can suppress the expression of tumor necrosis factor-α (TNF-α) and major histocompatibility complex (MHC II) by microglia in vivo or in vitro, thereby weakening their ability to present antigens to T cells [46]. A recent study further classified microglia into i-Mic, h-Mic, AP-Mic, and a-Mic [28]. AP-Mic represents a poor survival rate and exhibits metabolic characteristics of immunosuppressive macrophages. Therefore, our study selected AP-Mic as the main research object. Our results show that the BRMS1 + AP − Mic − C6 cluster exhibits a higher M2 macrophage-related signature score compared to other AP − Mic subtypes. In the local microenvironment, the presence of myeloid cells with phenotypic differences can lead to significant differences in cell-to-cell communication. Furthermore, we identified receptor-ligand pairs mediating cell type-specific communication in scRNA and observed changes in the quantity and intensity of communication between different AP − Mic subtypes mediated by anoikis and malignant cells. Among them, only BRMS1 + AP − Mic − C6 communicates with NPC-like/OPC-like malignant cells. In addition, we studied the bio-metabolism within six anoikis-related NMF cell clusters. The results show that BRMS1 + AP − Mic − C6, which has immunosuppressive properties, preferentially utilizes oxidative phosphorylation metabolism [47]. Finally, we explored the activity of transcription factors in six NMF cell clusters using SCENIC. In the BRMS1 + AP − Mic − C6 group, the activity of SPP1 is relatively higher. Interestingly, recent studies have shown that a subpopulation of SPP1 + TAMs and epidermal growth factor receptor (EGFR) amplification have been shown to be significantly associated with poor survival outcomes in patients. Moreover, infiltrating macrophages secrete SPP1,which maintains glioma cell survival and promotes angiogenesis [48, 49]. Given the complex intrinsic patterns of anoikis in TME cells, we conducted a comprehensive analysis to summarize the association between the scores of these subsets and their impact on prognosis and immune response. Our analysis through the TCGA and CGGA databases indicates that microglia with positive BRMS1 expression are likely to lead to poor prognosis and poor immune checkpoint blockade therapy response in GBM patients. Combined with the above analysis, BRMS1 seems to further promote the polarization of the M2 phenotype in microglia with certain immunosuppressive phenotypes, providing an immunosuppressive environment for the growth and development of tumor cells.

In our preliminary research, we mainly observed microglia and CD8 T cells, which exhibited different anoikis regulation patterns and engaged in complex communication with malignant GBM cells. Notably, we found that the inward and outward signaling patterns of MES-like cells are primarily mediated by the SPP1/CD44 signaling pathway. SPP1 is a multifunctional protein expressed in a variety of normal tissues, participating in cell–matrix and signal transduction processes. Numerous studies have linked SPP1 to pathological physiological conditions, including cancer. Emerging research has demonstrated the multifaceted roles of SPP1 in promoting tumor growth, regulating the tumor microenvironment, and its association with tumor metastasis and therapy resistance [50, 51]. In GBM, the β1 integrin-SPP1 signaling is positively correlated with higher macrophage density and lower overall survival rates [49]. Research by Tu et al. found that the GBM-secreted cytokine SPP1 promotes angiogenesis in GBM by upregulating the expression of prostate-specific membrane antigen (PSMA) in endothelial cells through the transcription factor HIF-1α [51]. As a well-characterized glioma invasiveness promoter, SPP1, secreted by glioma cells, can further promote macrophage infiltration and M2-like polarization, fostering immunosuppression and glioma growth [52]. CD44 is a complex transmembrane adhesion glycoprotein recognized as a marker of cancer stem cells and a key regulator of epithelial-mesenchymal transition (EMT), involved in tumor initiation, progression, and metastasis. Several single-cell sequencing studies have also shown that SPP1/CD44-mediated intercellular interactions between macrophages and cancer cells promote the remodeling of the ECM, forming a plastic suppressive microenvironment, and preventing lymphocyte infiltration into the tumor core, further reducing the efficacy of PD-L1 therapy [53, 54]. These results suggest that the role of SPP1/CD44-mediated intercellular interactions is likely a key factor in promoting the proliferation and invasion of GBM. To further validate the reliability of the scRNA-seq conclusions, we used SPOTlight to infer the spatial connections between BRMS1 + microglia and malignant cells. Both stRNA-seq and scRNA-seq indicate that BRMS1 + microglia may promote the malignant transformation of glioma cells through SPP1/CD44-mediated intercellular interactions.

Breast Cancer Metastasis Suppressor 1 (BRMS1) is a tumor metastasis suppressor gene discovered in breast cancer cells in 2000. Research has shown that it can inhibit tumor cell metastasis in various malignant tumors, significantly reducing the occurrence of metastatic foci without affecting tumor growth [55]. Recent clinical studies have confirmed that the expression of BRMS1 is reduced in a variety of malignant tumors, which seems to be associated with the invasion and metastasis of cancer cells and a better prognosis for patients [56]. Under hypoxic stress conditions, cancer cells dependent on BRMS1 exhibit increased sensitivity to anoikis, with a decreased survival rate [57]. Enhanced expression of BRMS1 can make non-small cell lung cancer cells sensitive to serum deprivation-induced apoptosis and increase cell viability [58]. Although gliomas share many attributes with metastatic cells, such as invasive growth, tumor cell migration, and neovascularization, they rarely metastasize outside the central nervous system [59]. A recent study showed that knockdown of BRMS1 in mutant P53 cells in vitro significantly reduced GBM cell growth and migration/invasion by inhibiting the NF-κB signalling pathway, suggesting that BRMS1 may be a potential therapeutic marker for mutant P53 GBM [60]. In our bioinformatics research and subsequent cell experiments, upregulation of BRMS1 in HMC3 cells promoted the proliferation, migration, and invasion of glioma cells, and inhibited apoptosis. The increased expression of BRMS1 in microglia is suggested to be linked to an unfavorable outcome in GBM patients, a finding that challenges the conclusions drawn from previous studies. Concurrently, Feldheim et al. reported a statistically significant relationship between the levels of BRMS1 mRNA and the proportion of cells staining positive for Ki67 (R = 0.36, *p* = 0.02). They propose that the regulation of BRMS1 expression could occur at various levels and during different phases, which might explain the discrepancies observed across multiple research findings [61].

## Conclusions

In this study, we successfully identified different cell subtypes in the tumor microenvironment mediated by anoikis using single-cell RNA sequencing analysis, as well as their significant predictive value for prognosis and immune therapy response in glioblastoma. Microglia with high BRMS1 expression provides a new perspective for a deeper understanding of the immunosuppressive microenvironment in GBM and could serve as potential therapeutic targets for the future. The underlying mechanisms of BRMS1 in gliomas remain to be fully elucidated and represent a promising focus for future research.

## Supplementary Information

Below is the link to the electronic supplementary material.

Supplementary file1 (TIF 5776 KB)

Supplementary file2 (TIF 3934 KB)

Supplementary file3 (TIF 9593 KB)

Supplementary file4 (XLS 20 KB)

## Data Availability

The datasets supporting the conclusions of this article are included within the article.
