# Supplementary figures and images for: Exploring the prognostic value of BRMS1 + microglia based on single-cell anoikis regulator patterns in the immunologic microenvironment of GBM
Source: J Neurooncol. 2024 Aug 15;170(1):101–17. doi: 10.1007/s11060-024-04781-5 (PMC11447114; doi:10.1007/s11060-024-04781-5)

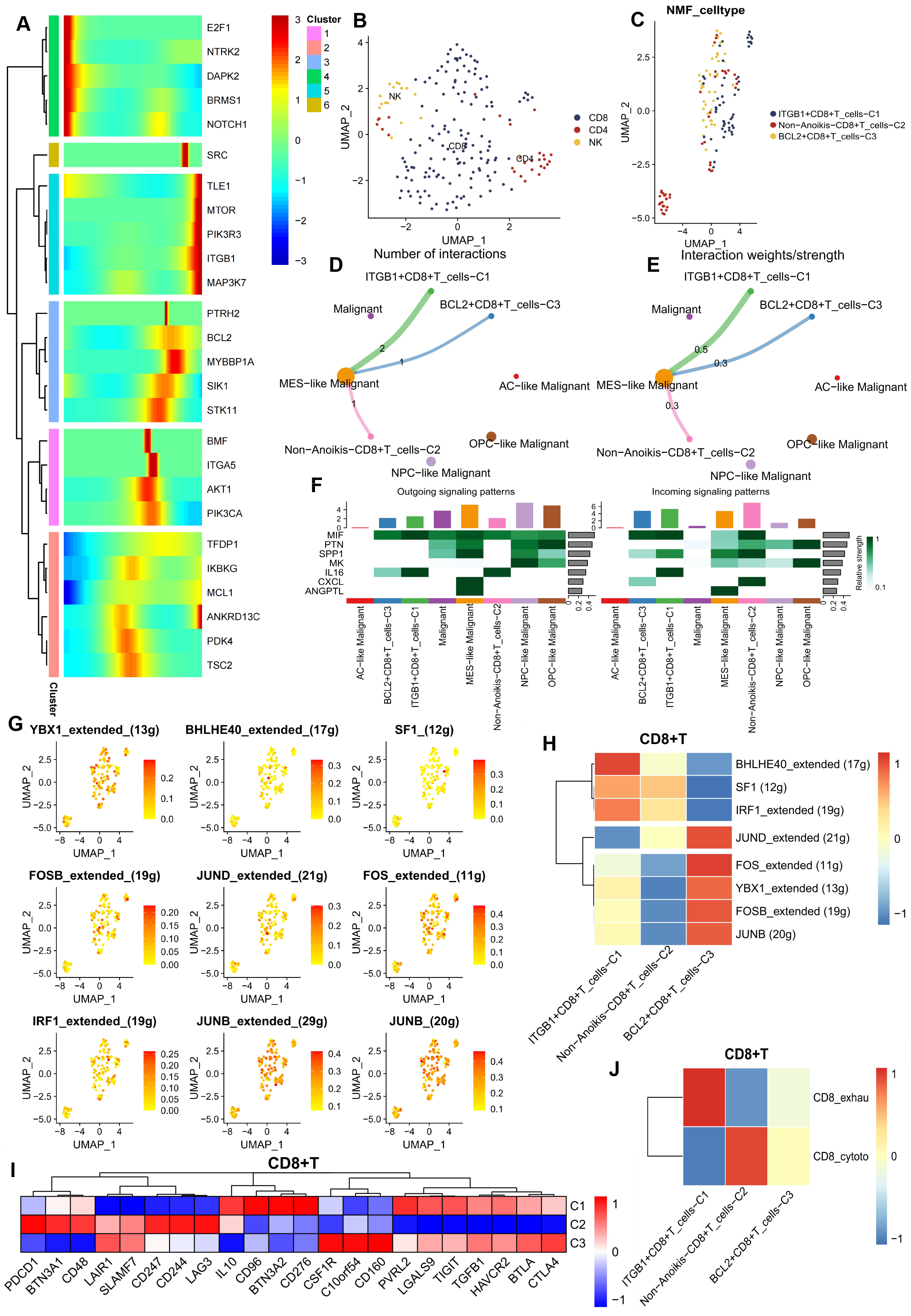

Supplement: Supplementary file 1 — Supplementary file1 (TIF 5776 KB) [file 11060_2024_4781_MOESM1_ESM.tif]

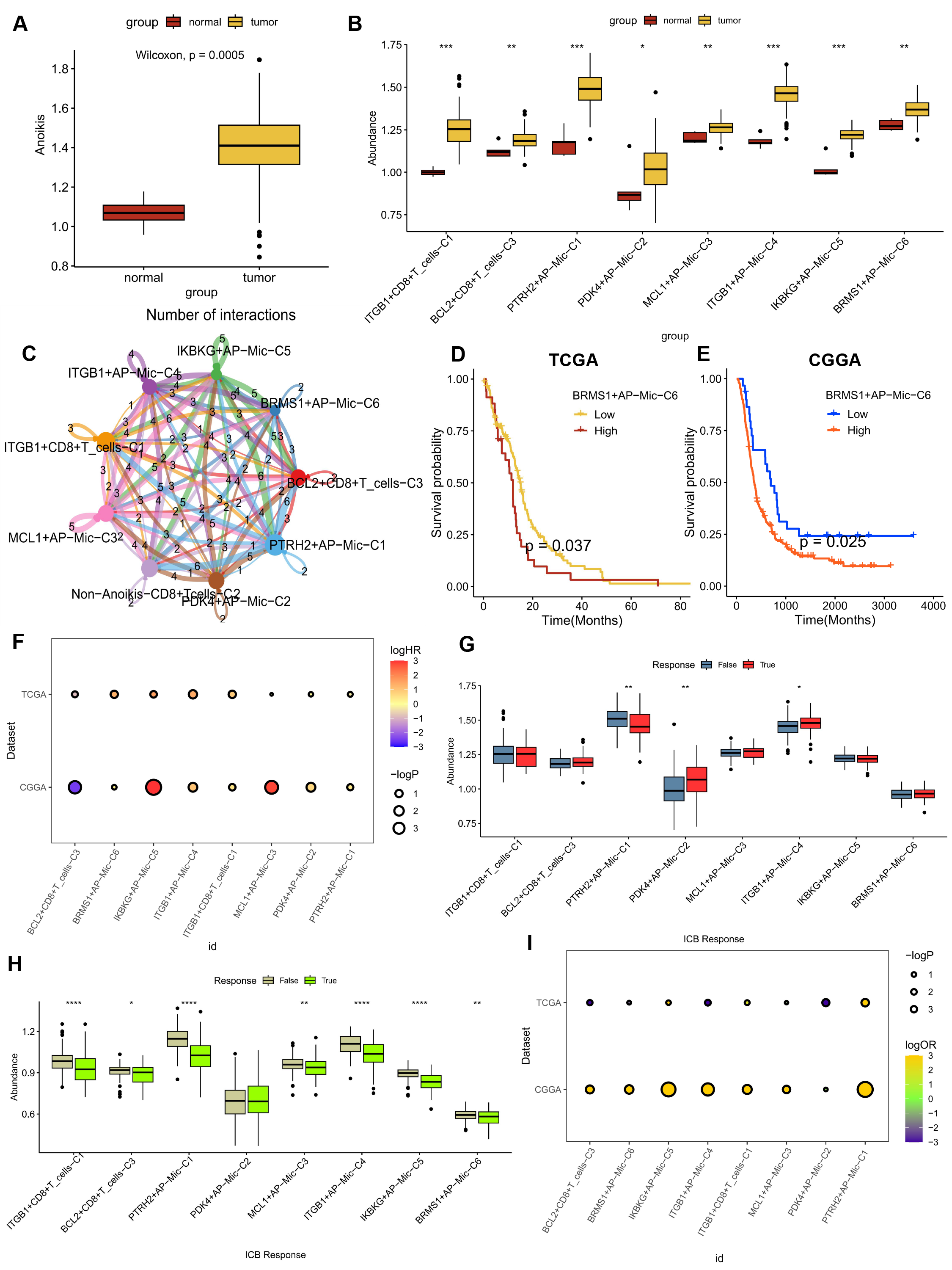

Supplement: Supplementary file 2 — Supplementary file2 (TIF 3934 KB) [file 11060_2024_4781_MOESM2_ESM.tif]

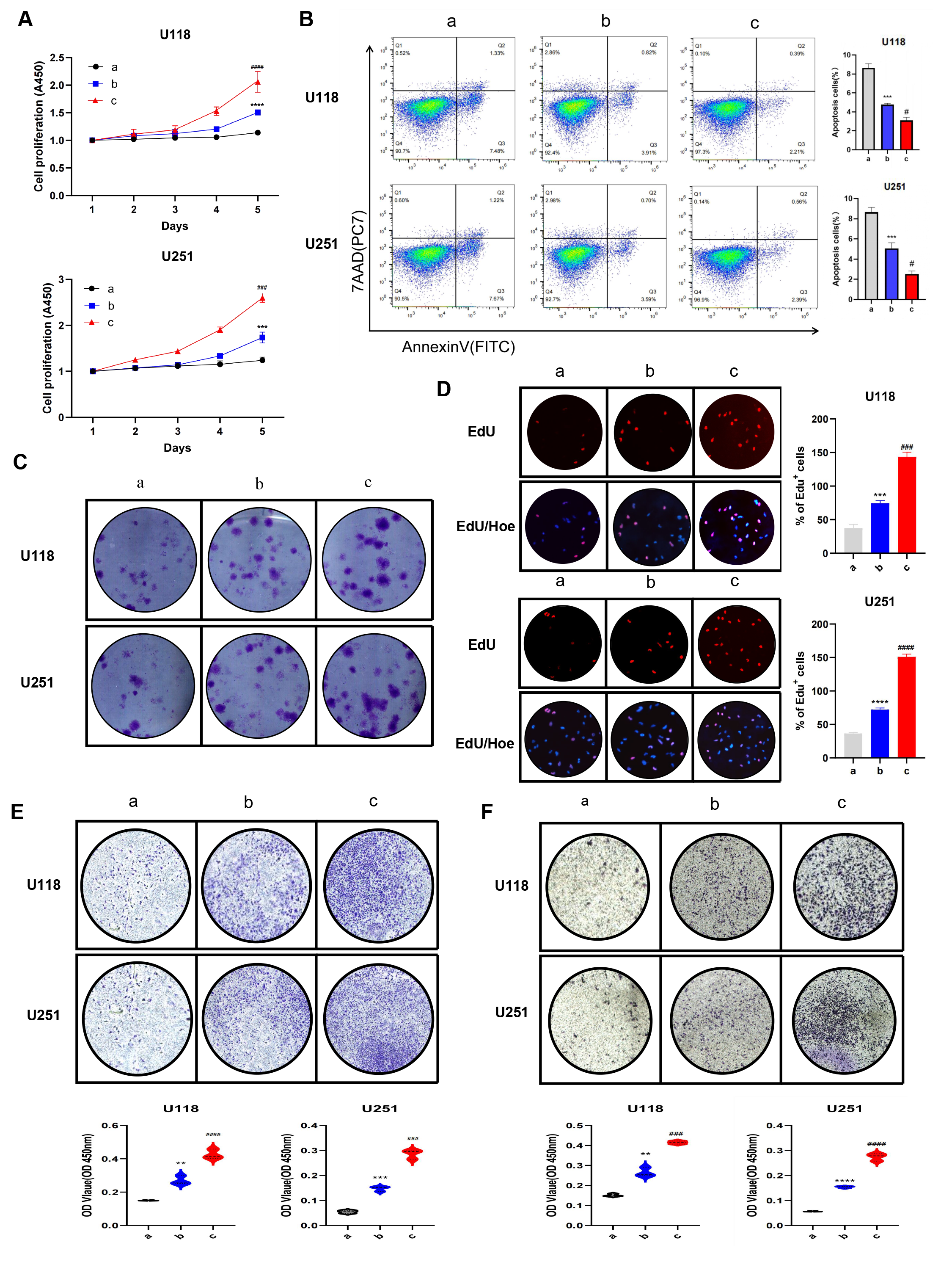

Supplement: Supplementary file 3 — Supplementary file3 (TIF 9593 KB) [file 11060_2024_4781_MOESM3_ESM.tif]
